# Topical Application of KAJD Attenuates 2,4-Dinitrochlorobenzene-Induced Atopic Dermatitis Symptoms Through Regulation of IgE and MAPK Pathways in BALB/C Mice and Several Immune Cell Types

**DOI:** 10.3389/fphar.2019.01097

**Published:** 2019-09-19

**Authors:** Se Hyang Hong, Jin Mo Ku, Hyo In Kim, Tai Young Kim, Hye Sook Seo, Yong Cheol Shin, Seong-Gyu Ko

**Affiliations:** ^1^Department of Preventive Medicine, College of Korean Medicine, Kyung Hee University, Seoul, South Korea; ^2^Department of Science in Korean Medicine, Graduate School, Kyung Hee University, Seoul, South Korea

**Keywords:** atopic dermatitis, mast cell, splenocyte, macrophage, IgE, CD4+ T cells, MAPK pathway

## Abstract

Atopic dermatitis (AD) is a frequent skin complication that is caused by unknown reasons. KHU-ATO-JIN-D (KAJD) is a new drug aimed at AD composed of a mixture of extracts from six plants known to have anti-inflammatory and antiallergic effects. This study investigated whether KAJD alleviates 2,4-dinitrochlorobenzene (DNCB)-induced AD in BALB/c mice and several immune cell types. We applied KAJD to DNCB-induced AD-like skin lesions in BALB/c mice, phorbol myristate acetate/ionomycin-stimulated human mast cells (HMC-1), and lipopolysaccharide (LPS)-stimulated macrophages and splenocytes. Histological, ELISA, PCR, and Western blot experiments were performed. The application of KAJD significantly attenuated the lesion severity and skin thickness and inhibited the infiltration of inflammatory cells, mast cells, and CD4+ T cells into the sensitized skin of mice. Reduced leukocyte numbers and proinflammatory cytokine and IgE levels were also observed in the sera of KAJD-treated mice. Moreover, *in vitro* studies demonstrated that KAJD treatment reduced the LPS-induced expression of proinflammatory cytokines and nitric oxide (NO) production in RAW 264.7 cells. The regulation of IL-4 and IL-6 mRNA and MAPK pathways was also detected in agonist-induced isolated splenocytes and HMC-1 cells by the addition of KAJD. Taken together, our results demonstrate that KAJD inhibits the development of DNCB-induced AD in BALB/c mice and in several immune cell types, suggesting that KAJD might be a useful therapeutic drug for the treatment of AD.

## Introduction

Atopic dermatitis (AD) is a common chronic inflammatory skin disease and often causes other allergic disorders, such as food allergies, asthma, and rhinitis (Leung and Bieber, 2003; Spergel and Paller, 2003). Atopic dermatitis is characterized by eczematous skin lesions, pruritic folliculitis, and increased serum immunoglobulin E (IgE) levels, which are associated with infiltration of inflammatory cells, such as lymphocytes, macrophages, eosinophils, and mast cells (Rousset et al., 1991; Simon et al., 2004; Marsella et al., 2011).

Mast cells are activated through Th2 cytokines or cross-linking of their high-affinity surface receptors with IgE (FcεRI), thereby leading to cell degranulation and production of inflammatory mediators (Novak et al., 2004; Ong and Leung, 2006; Theoharides et al., 2012). Atopic dermatitis is also associated with elevated levels of Th2 cytokines, IL-4, IL-5, and IL-13 in blood (Homey et al., 2006) and infiltration of CD4+ T cells into AD skin lesions (Leung and Bieber, 2003). Corticosteroids are one of the most common topical medications for treating AD, but their long-term use produces undesired side effects (Lubach et al., 1989; Blume-Peytavi and Wahn, 2011). Although topical immunosuppressants such as tacrolimus (TAC) have been introduced as an alternative steroid-free anti-inflammatory agent for the safe and effective treatment of patients with AD (Akhavan and Rudikoff, 2008), several side effects of TAC, such as itching, burning sensation of the skin, flu-like symptoms, headache, cough, and burning eyes, have also been reported (Hanifin et al., 2005). Consequently, the development of new useful drugs that efficiently manage AD and have reduced side effects is urgently needed.

In this study, we introduce KHU-ATO-JIN-D (KAJD), which is a mixture of extracts from six plants (*Phellodendri cortex, Schizonepetae spica, Sophorae radix, Glycyrrhizae radix, Liriopis tuber*, and *Radix rehmanniae* Exsiccat), as a novel medication for treating AD. Each extract has been shown to demonstrate anti-inflammatory and antiallergic activity (Ho-Sub et al., 2001; Lee et al., 2005; Shin et al., 2008; Ra et al., 2011; Han et al., 2012; Choi et al., 2014). We used BALB/c mice as a model animal because they exhibited symptoms very similar to those of human AD, including an increased IgE level and chronic dryness (Dearman et al., 1995), and 2,4-dinitrochlorobenzene (DNCB) was used to induce AD in BALB/c mice (Kim et al., 2014; Wang et al., 2015; Yoon et al., 2015).

In the present study, we sought to explore the therapeutic effects of KAJD on DNCB-induced AD-like symptoms in BALB/c mice. In addition, we investigated the anti-inflammatory effects of KAJD on macrophages, mast cells, and splenocytes. Our results demonstrate that KAJD inhibits the development of DNCB-induced AD-like symptoms, suggesting that KAJD might be a useful therapeutic drug for the treatment of AD.

## Materials and Methods

### Preparation of KAJD and HPLC Analysis

**Figure 1:**
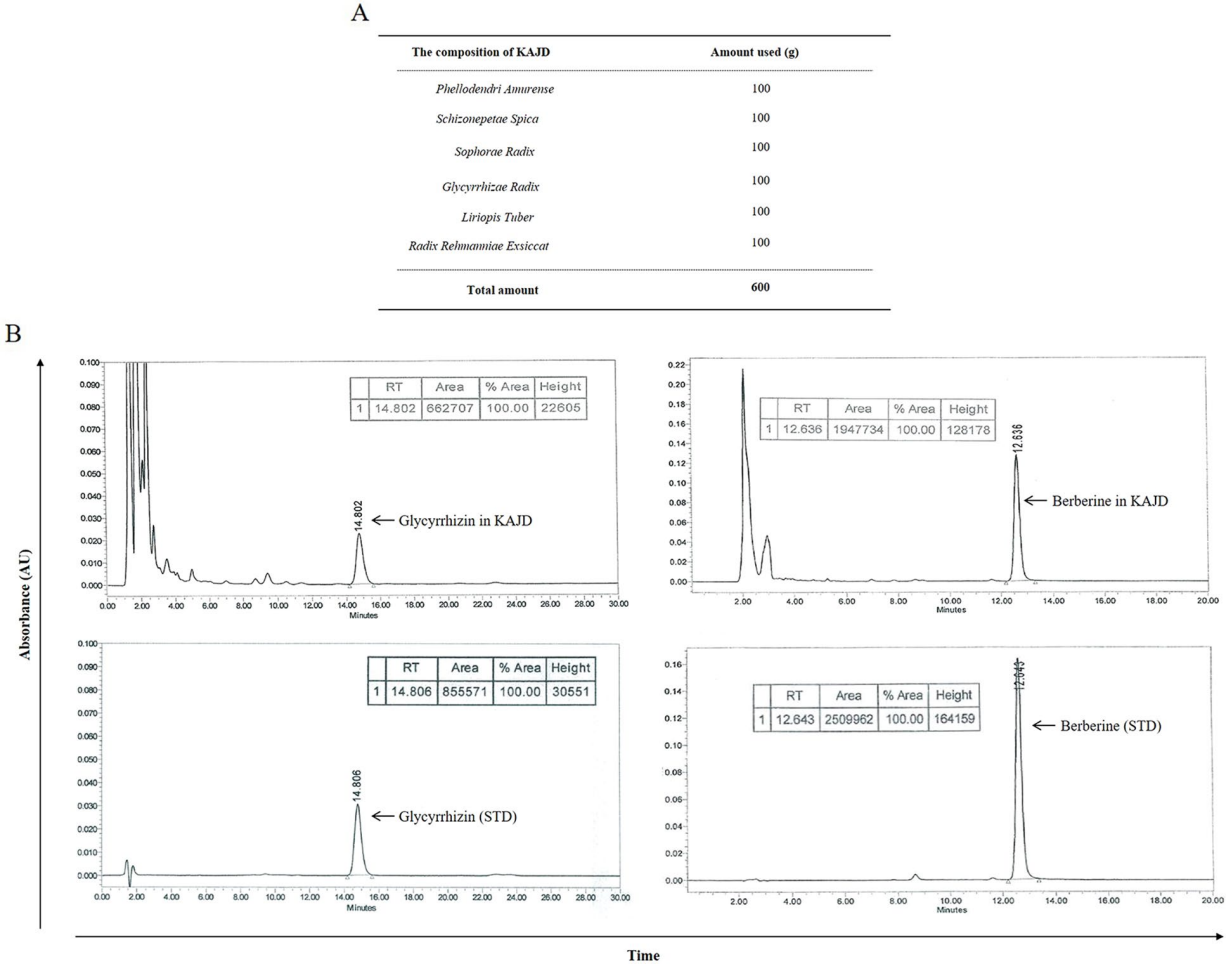
HPLC profile of KAJD. **(A)** Composition of KAJD. **(B)** HPLC identification of components in KAJD. Glycyrrhizin and berberine in KAJD were detected at 14.802 min and 12.636 min, respectively. AU, Arbitrary units; STD, Standard.

KHU-ATO-JIN-D, prepared by Hanpoong Pharm and Foods Company (Jeon-ju, Korea) in accordance with good manufacturing practice (GMP) protocols, is a water-extracted brown-colored mixture composed of *Phellodendri amurense, Schizonepetae spica, Sophorae radix, Glycyrrhizae radix, Liriopis tuber*, and *Radix rehmanniae* Exsiccat (**Figure 1A**). High-performance liquid chromatography (HPLC) was performed to confirm the characteristics of herbal mixtures, including each component (Hanpoong Pharm and Foods Company) (**Figure 1B**). KHU-ATO-JIN-D was passed through a 0.2-μm membrane filter, and 20 μl aliquots of the filtrate were injected into the HPLC. The Hypersil GOLD C18 analytical column (250 mm × 4.6 mm; particle size 5 μm) was used, and the mobile phases consisted of solvent A (DI water) and solvent B (acetonitrile), which were applied at a flow rate of 1 ml/min. The column temperature was set at 30°C. The injection volume was 10 μl, and content analysis was performed at 280nm. Glycyrrhizin and berberine were used as standard compounds (Sigma, St. Louis, MO, USA) at a concentration of 1mg/ml to identify the peaks [**Figure 1B** (lower panel)]. The total glycyrrhizin and berberine contents in the extracts were 8.05 and 4.8 mg/g, respectively (**Figure 1B** (upper panel)). More information on the KAJD production processes can be obtained upon request from Hanpoong Pharm and Foods Company (http://hpeng.hanpoong.co.kr/). The KAJD cream was made by ATEC & Co. (Seoul, Korea) in accordance with cosmetic guidelines on good manufacturing practice (CGMP) protocols for animal experiments. More information on the KAJD cream production processes can be obtained upon request from ATEC & Co. (http://www.atecltd.com/).

### Animal Studies

Six-week-old male BALB/c mice (20 ± 2 g) were purchased from Orient (Sung-nam, Korea) and randomly assigned into four groups (no treatment, DNCB, DNCB + TAC, DNCB + KAJD); each group included five mice. All mice were housed in a pathogen-free environment and provided *ad libitum* access to food and water. Our methods were executed in accordance with relevant guidelines, and regulations and procedures involving mice were approved by the animal care center of Kyung Hee University (approval number KHUASP (SE)-14-014).

### Sensitization and Treatment

To promote the development of AD-like skin lesions, the mouse back skin was shaved and sensitized twice a week by applying 100 µl of 2% DNCB in acetone using 1 × 1 cm patches and followed with a second challenge of 100 µl of 0.2% DNCB twice a week. Control mice were painted with acetone alone. Next, KAJD or TAC was applied to the sensitized mouse skin regularly for 2 weeks. On day 28, all mice were sacrificed by CO_2_ inhalation, and blood samples were collected. The method was performed as described previously (Ku et al., 2017).

### Clinical Skin Score

Clinical observation to assess changes in the skin of BALB/c mice was performed once a week for 4 weeks. The severity of AD-like dorsal skin lesions was evaluated based on four symptoms, including erythema/hemorrhage, scarring/dryness, edema, and excoriation/erosion; each of which was graded on a scale from 0 to 3 (none, 0; mild, 1; moderate, 2; severe, 3). The clinical skin score was defined as the sum of the individual scores and ranged from 0 to 12.

### Skin Thickness and Spleen Weight

Before sacrifice, the epidermal thickness at three different mouse back skin sites was measured with a digital caliper (Mitutoyo, Kawasaki, Japan). The spleens from all mice were removed and weighed immediately.

### Histological Analysis

Portions of the skin biopsies were fixed in 4% paraformaldehyde (PFA) and embedded in Frozen Section Compound (FSC22 Clear, Surgipath, Leica Biosystem, Wetzlar, Germany) on dry ice. Skin sections of 20 µm thickness were cut and stained with hematoxylin and eosin (H&E) to detect inflammatory cells or with toluidine blue (T. B) to detect mast cells. The stained tissues were examined under a light microscope (Olympus, Tokyo, Japan), and the inflammatory or mast cells were counted in 10 random high-power field (HPF) sections at 400× magnification.

### Immunohistochemistry

We performed immunohistochemical analysis to detect CD4+ lymphocytes using the anti-CD4+ antibody. Briefly, the skin sections were hydrated, and after heating, the sections were incubated with 3% hydrogen peroxide (PBS) for 15 min to prohibit endogenous peroxidase activity in blood cells. Next, the skin sections were treated with a blocking solution [5% bovine serum albumin (BSA) in PBS] for 1 h at room temperature. The sections were then treated with a mouse monoclonal CD4+ antibody overnight at 4°C and incubated with secondary biotinylated anti-rabbit IgG for 1 h at room temperature. The skin sections were next treated with an avidin–biotin horseradish peroxidase (HRP) complex (VECTASTAIN ABC Kit, Vector Labs, Burlingame, CA, USA) for 30 min at 4°C and stained with diaminobenzidine tetrachloride (DAB) as a substrate. The sections were counterstained with hematoxylin, mounted with an aqueous mounting solution (Permount, Fisher Scientific, Waltham, MA, USA), and coverslipped. The skin sections were analyzed using an Olympus microscope, and images were captured using a digital video camera. The CD4+ lymphocytes were counted in 10 random HPF sections at 400× magnification.

### Blood Analysis

Whole blood samples were collected from mice by cardiac puncture under anesthesia and placed in Vacutainer TM tubes containing EDTA (BD Science, NJ, USA). To determine the hematological parameters [white blood cells (WBC), lymphocytes, monocytes, eosinophils, basophils, and neutrophils] of the blood samples, a HEMAVET 950 hematology analyzer (Drew Scientific, Inc., Oxford, Dallas, TX, USA) was used in accordance with the manufacturer’s recommendation.

### Enzyme-Linked Immunosorbent Assay

The levels of cytokines and total IgE in the sera of mice and the expression levels of cytokines in cell lines, including RAW264.7 cells, HMC-1 cells, and splenocytes, were measured by sandwich ELISA using the BD Pharmingen mouse or human ELISA Set (Pharmingen, San Diego, CA, USA). Briefly, plates were coated with a capture antibody in ELISA coating buffer (Sigma, Louis, MO, USA) and incubated overnight at 4°C. The plates were washed with PBS-Tween 20 (0.05%) and subsequently blocked [10% fetal bovine serum (FBS) in PBS] for 1 h at 20°C. Serial dilutions of standard antigen or sample in dilution buffer (10% FBS in PBS) were added to the plates and incubated for 2 h at 20°C. After washing, biotin-conjugated anti-mouse IgE and streptavidin-HRP conjugate (Sav-HRP) were added to the plates and incubated for 1 h at 20°C. Finally, tetramethylbenzidine (TMB) substrate solution was added to the plates for 15 min in the dark, followed by the addition of 2N H_2_SO_4_ to stop the reaction. Optical densities were measured at 450 nm on an automated ELISA reader (Versa Max, Molecular Devices, Sunnyvale, CA, USA).

### Cell Culture

The RAW 264.7 murine macrophage cell line and the human mast cell (HMC-1) line were purchased from the Korea Cell Line Bank (Seoul, Korea). Each cell line was grown in Dulbecco’s modified Eagle’s medium (DMEM, Welgene, Daegu, Korea) or Iscove’s Modified Dulbecco’s Medium (IMDM, Welgene, Daegu, Korea) supplemented with 10% heat-inactivated FBS (Welgene, Daegu, Korea) and 1% antibiotics (Ab, Welgene, Daegu, Korea) at 37°C and 5% CO_2_.

### Splenocyte Isolation

Splenic cell suspensions from BALB/c mice were prepared in RPMI-1640 medium (containing 10% FBS, 1% Ab, and 0.05 mM mercaptoethanol) by homogenization under aseptic conditions. The contaminating red blood cells were removed using red blood cell lysis buffer (Sigma, St. Louis, MO, USA). The suspension was centrifuged and resuspended in complete RPMI-1640. The isolated spleen cells were maintained at 37°C in an incubator humidified at 5% CO_2_.

### Cell Viability Assay

RAW 264.7 cells, HMC-1 cells, and splenocytes (1× cells/well) were plated in 96-well culture plates and incubated for 24 h. RAW264.7 cells and splenocytes were treated with 1 µg/ml LPS, and HMC-1 cells were treated with 5 ng/ml phorbol-12-myristate (PMA) and 500 ng/ml ionomycin (Ion) in the presence or absence of various concentrations of KAJD. After 24 h of incubation, 10 μl of water-soluble tetrazolium (WST) solution was added to each well of the plate, which was incubated in the dark at 37°C for another 1 h. Optical density was measured at 450 nm using an ELISA Plate Reader (Versa Max, Molecular Devices, Sunnyvale, CA, USA).

### Detection of Nitric Oxide

Nitric oxide (NO) production was measured in the RAW 264.7 culture supernatant using a Griess Reagent Kit (Promega, Madison, WI, USA). In brief, 150 µl of culture supernatant was transferred to a 96-well plate and then mixed with 150 µl of Griess reagent solution. The mixtures were then incubated for 30 min at room temperature, and optical density was determined at 570 nm using a microplate reader.

### Reverse Transcription-Polymerase Chain Reaction

Cells were harvested by centrifugation, and the pellet was washed with ice-cold PBS. RNA was isolated from the pellet using an easy-BLUE RNA Extraction Kit (iNtRON Biotech, Sungnam, Korea) according to the manufacturer’s instructions. The quantity of the isolated RNA was measured using the NanoDrop ND-1000 Spectrophotometer (NanoDrop Technologies Inc., Wilmington, DE, USA). cDNA was synthesized from 2 µg of total RNA using a cDNA Synthesis Kit (TaKaRa, Otsu, Shinga, Japan). RT-PCR was performed in a 20 µl reaction mixture consisting of DNA template, 10 pM of each gene-specific primer, 10× Taq buffer, 2.5 mM dNTP mixture, and 1 unit of Taq DNA polymerase (Takara, Otsu, Shinga, Japan). The primer sequences used for human and/or mouse IL-4, IL-6, and GAPDH are shown in **Table 1**.

**Table 1:** PCR primer sequences.

Primer type	Primer name	Primer sequence
Mouse	IL-4	F: 5’-TCG GCA TTT TGA ACG AGG TC-3’
		R: 5’-GAA AAG CCC GAA AGA GTC TC-3’
	IL-6	F: 5’-CAA GAG ACT TCC ATC CAG TTG C-3’
		R: 5’-TTG CCG AGT TCT CAA AGT GAC-3’
	GAPDH	F: 5’-GAG GGG CCA TCC ACA GTC TTC-3’
		R: 5’-CAT CAC CAT CTT CCA GGA GCG-3’
Human	IL-4	F: 5’-TGC CTC CAA GAA CAC AAC TG-3’
		R: 5’-CTC TGG TTG GCT TCC TTC AC-3’
	IL-6	F: 5’-AAC CTT CCA AAG ATG GCT GAA-3’
		R: 5’-CAG GAA CTG GAT CAG GAC TTT-3’
	GAPDH	F: 5’-CGT CTT CAC CAC CAT GGA GA-3’
		R: 5’-CGG CCA TCA CGC CAC AGT TT-3’

### Western Blot Analysis

Cells were harvested, and cell pellets were incubated in one volume of lysis buffer containing 20 mM Tris–HCl (pH 7.5), 150 mM NaCl, 1 mM EDTA, 1 mM Na_2_EDTA, 1 mM EGTA, 1% NP-40, 1% sodium deoxycholate, 1 mM Na_3_VO_4_, 1 mM DTT, 1 mM NaF, 1 mM PMSF, and propidium iodide (PI) cocktail on ice for 30 min, centrifuged at 13,000 rpm for 20 min at 4°C, aliquoted to 20 µl, and transferred onto nitrocellulose membranes (Protran Nitrocellulose Membrane, Whatman, UK). The membranes were blocked with 5% nonfat milk and probed with specific primary antibodies. After washing, the membranes were incubated with diluted enzyme-linked secondary antibodies, and the protein bands were detected using an EZ-Western Chemiluminescence Detection Kit and visualized by exposing the membranes to X-ray films. In a parallel experiment, cytoplasmic and nuclear proteins were extracted using NE-PER^®^ Nuclear and Cytoplasmic Extraction Reagents (Pierce Biotechnology, Rockford, IL, USA) according to the manufacturer’s instructions. Each protein was appropriately detected with the following antibodies: anti-p-EKR1/2, p-P38, and p-JNK antibodies (all purchased from Santa Cruz Biotechnology [Santa Cruz, CA, USA]) and anti-GAPDH antibodies (purchased from Cell Signaling Technology [Danvers, MA, USA]).

### Statistical Analysis

All quantitative data derived from this study were analyzed statistically. The results are expressed as the mean ± standard deviation (SD) or mean ± SEM of at least three separate tests. Statistical significance was determined using one-way analysis of variance followed by the Tukey–Kramer multiple comparisons post-test to analyze differences between groups. P < 0.05 was considered to indicate a statistically significant difference, and P < 0.05, <0.01, and <0.001 are assigned respective symbols in the figures. All experiments were performed at least three times. All statistical analyses were performed using PRISM software (version 5.0; GraphPad Software Inc., La Jolla, CA, USA).

## Results

### Topical Application of KAJD Suppresses DNCB-Induced AD in Mice

**Figure 2:**
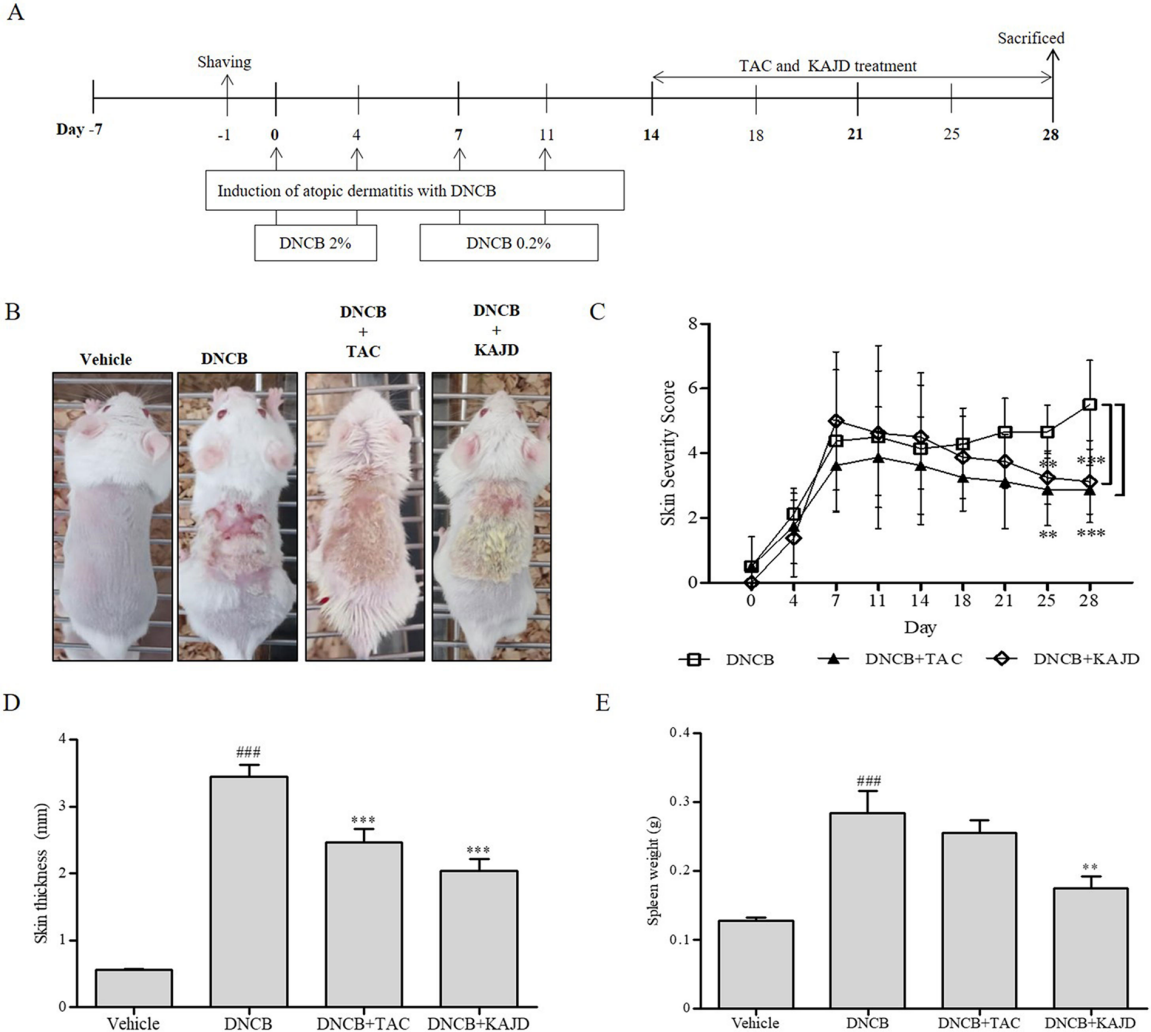
KAJD suppresses DNCB-induced atopic dermatitis-like symptoms. **(A)** BALB/c mice were sensitized with the sequential application of 2% and 0.2% DNCB for 2 weeks. KAJD or tacrolimus was regularly applied to the sensitized skin for an additional 2 weeks. **(B)** Photographs were taken at the end of the experiment to show the appearance of skin lesions (n=8). **(C)** Skin severity scores of AD-like skin lesions in BALB/c mice. The total score is the sum of individual scores determined based on the symptoms of erythema/hemorrhage, edema, scaling/dryness, and excoriation/erosion. **(D**, **E)** The dorsal skin thickness **(D)** and spleen weights **(E)** of BALB/c mice were measured at the end of treatment. ^###^ P < 0.001 compared to the vehicle group. **P < 0.01 and ***P < 0.001 compared to the DNCB-stimulated group. Vehicle, no treatment; DNCB, 2,4-dinitrochlorobenzene; TAC, tacrolimus.

To investigate the effects of KAJD on DNCB-induced AD-like symptoms, BALB/c mice were first sensitized with multiple applications of DNCB on their skin and then treated with KAJD or TAC ointment as a control on a regular basis for 2 weeks (**Figure 2A**). All mice developed significant skin hypersensitivity reactions after the DNCB application, showing severe erythema/hemorrhage, scarring/dryness, edema, and excoriation/erosion (**Figure 2B**). Scoring of the skin lesion severity revealed that the wounds were very severe and lasted until the mice were sacrificed. However, subsequent topical application of KAJD or TAC on the sensitized skin markedly reduced the skin severity. On day 28, the clinical scores of 3.13±1.27 in the KAJD-treated group and 3.29±0.76 in the TAC-treated group were significantly lower than that of 5.5±1.38 in DNCB-treated mice (**Figure 2C**). Moreover, the skin thickness was increased by DNCB and markedly reduced by the application of KAJD or TAC (**Figure 2D**). It is well known that the weights of some immune organs increase in response to the topical application of agents that have allergenic or sensitizing potential (Stahlmann et al., 2006; Bae et al., 2010). Similarly, the increased spleen weight in DNCB-treated mice was slightly decreased in response to KAJD or TAC treatment (**Figure 2E**). Notably, KAJD exhibited a greater potential for ameliorating the DNCB-induced increase in skin thickness and spleen weight than TAC. Taken together, these results demonstrate that KAJD effectively attenuates DNCB-induced AD-like symptoms and is superior to TAC in some ways. We also monitored the mouse body weight changes and food intake twice a week throughout the study. Mice treated with DNCB did not show any changes in food intake, but their body weight was decreased by 15% compared with that of vehicle-treated mice. Treatment with KAJD or TAC did not markedly alter the index of DNCB-treated mice (**Supplementary Figure 1**).

### KAJD Decreases the DNCB-Induced Infiltration of Inflammatory Cells, Mast Cells, and CD4+ T Cells Into AD Skin Lesions

**Figure 3:**
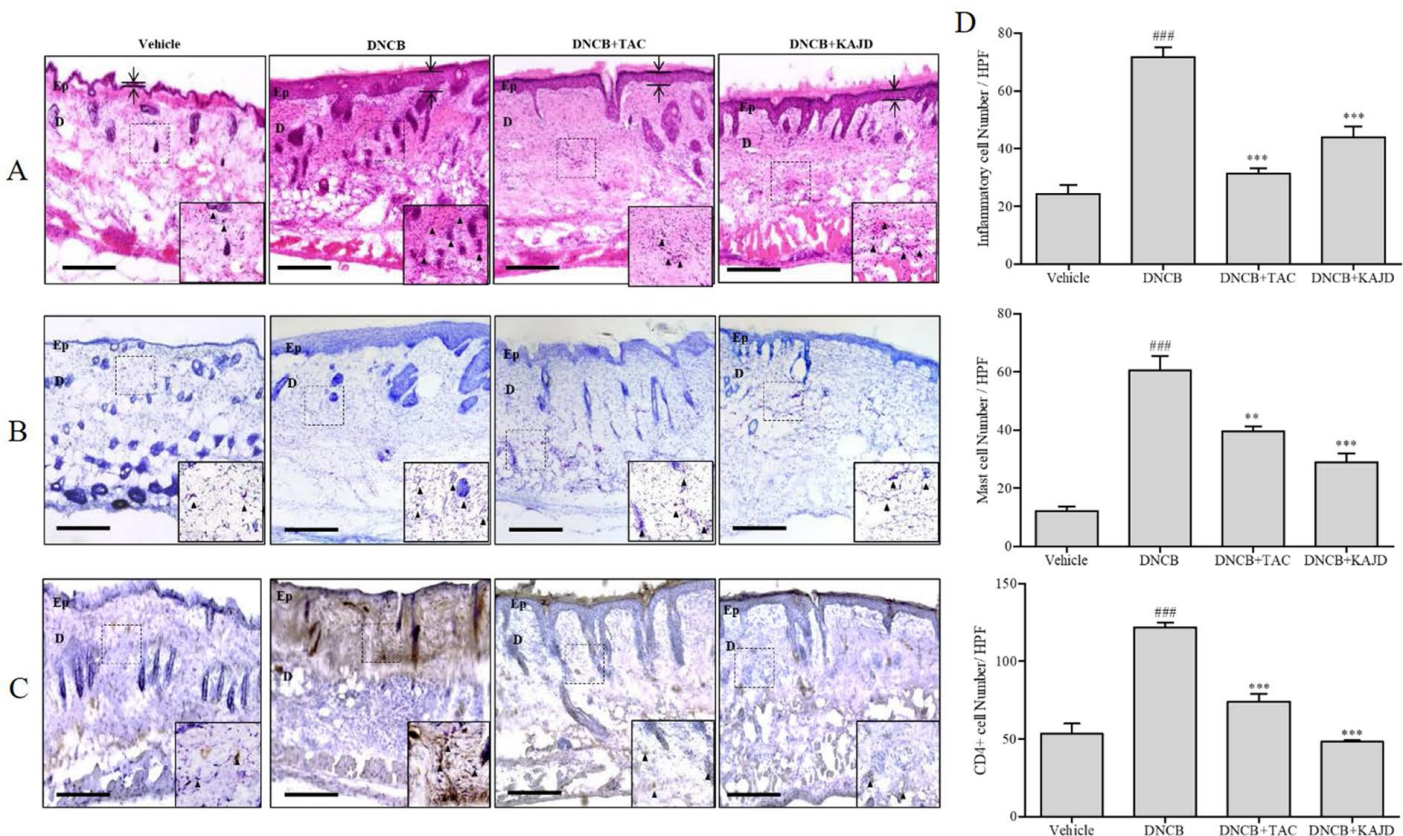
KAJD reduces the dermal infiltration of inflammatory cells and mast cells in DNCB-treated BALB/c mice. **(A**, **B**, and **C)** Dorsal skin sections were stained with hematoxylin and eosin to show inflammatory cells as indicated by the purple spot **(A)**, stained with toluidine blue to show mast cells as indicated by the purple spot **(B)**, or stained with diaminobenzidine tetrachloride (DAB) and hematoxylin to show CD4+ T cells as indicated by the brown spot **(C)**. The arrows and bars indicate the thickness of the epidermis. **(D)** The stained inflammatory cells (upper panel), mast cells (middle panel), and CD4+ cells (lower panel) were counted, and the results are presented in a graph. Sections were evaluated using a microscope at an original magnification of 400×. The data are shown as the mean ± SEM. ^###^ P < 0.001 compared to the vehicle group. **P < 0.01 and ***P < 0.001 compared to the DNCB-stimulated group. Bar = 50 µm. Vehicle, no treatment; DNCB, 2,4-dinitrochlorobenzene; TAC, tacrolimus; Ep, epidermis; D, dermis.

Next, we examined the recruitment of inflammatory cells to sensitized skin by H&E staining. While repeated cutaneous application of DNCB induced the infiltration of inflammatory cells into both the epidermis and dermis, subsequent application of KAJD to the allergic skin inhibited their recruitment (**Figure 3A**). Mast and CD4^+^ T cells are well known to be involved in allergenic reactions and inflammation (Ahmadzadeh et al., 2001; Liu et al., 2011). T. B staining showed that DNCB increased the number of dermal mast cells on the treated skin, and this increase was attenuated by KAJD (**Figure 3B**). Moreover, DNCB increased the number of CD4^+^ T cells, while KAJD decreased this number in the skin epidermis and dermis (**Figure 3C**). Bar graphs indicate the average number of cells counted in a random field of view (**Figure 3D**). Notably, although TAC also reduced the recruitment of inflammatory cells, mast cells, and CD4^+^ T cells to the skin, KAJD was more effective for the inhibition of mast cell and CD4^+^ T cell recruitment than TAC.

### KAJD Suppresses the DNCB-Induced Increase in Mouse WBCs

**Figure 4:**
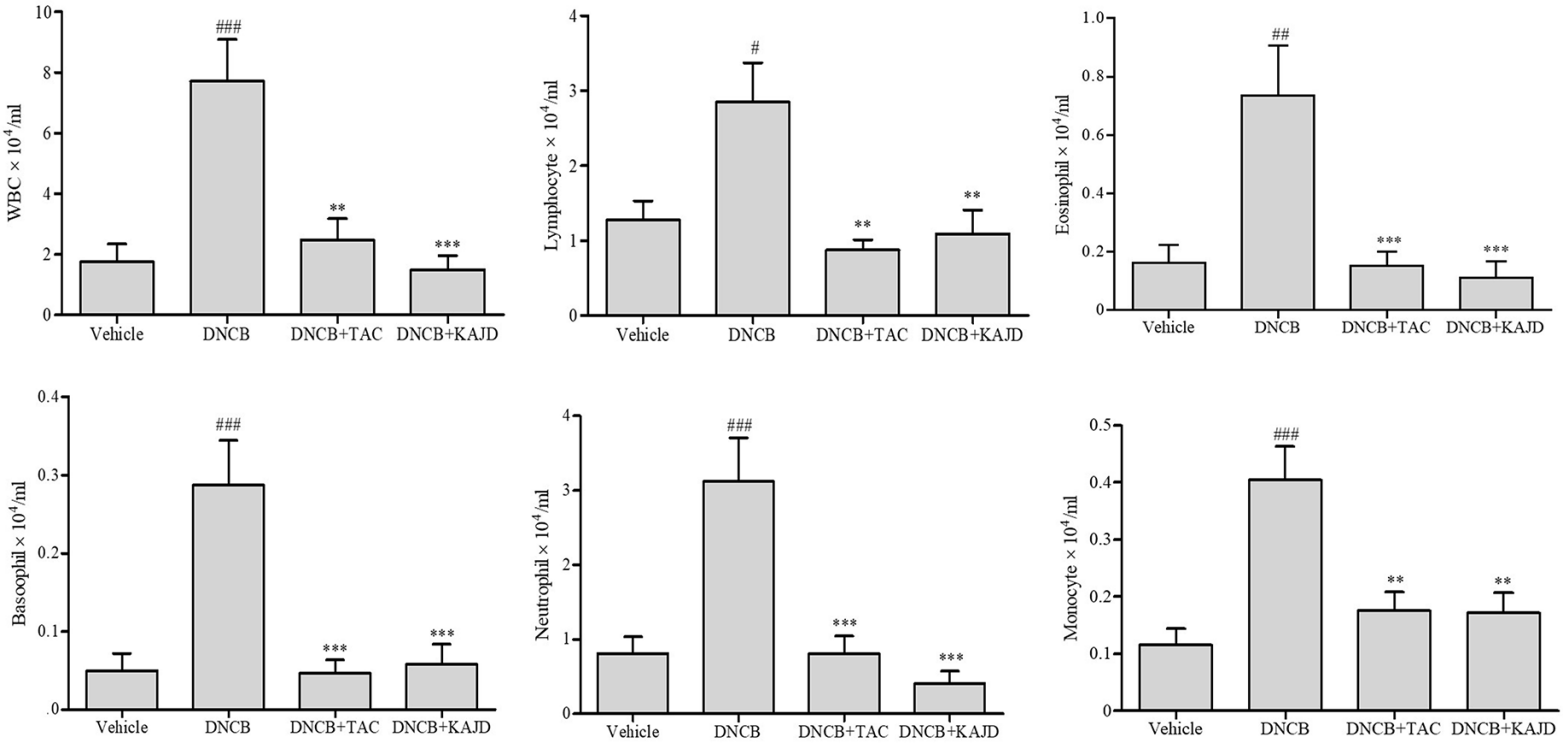
KAJD suppresses the number of WBCs. WBCs in each group of mice were analyzed for subtype cell numbers using a HEMAVET hematology analyzer. The data are presented as the mean ± SEM. ^#^P < 0.05 and ^###^P < 0.001 compared to the vehicle group. **P < 0.01 and ***P < 0.001 compared to the DNCB-stimulated group. Vehicle, no treatment; DNCB, 2,4-dinitrochlorobenzene; TAC, tacrolimus.

Since the numbers of most types of WBCs are increased in patients with AD (Lubach et al., 1989; Hanifin et al., 2005), we determined the number of WBCs in mouse blood using a HEMAVET 950 Hematology Analyzer. In mice treated with DNCB, the total number of WBCs was increased up to 3.5-fold compared with that in vehicle-treated mice, but this number was dramatically reduced by treatment with KAJD or TAC (**Figure 4**). Counting each subtype of WBCs, including granulocytes (neutrophils, basophils, eosinophils), monocytes, and lymphocytes, showed that KAJD and TAC suppressed the DNCB-induced increases in the numbers of each WBC cell type to similar extents. In particular, both KAJD and TAC effectively decreased the eosinophil cell count, which is correlated with the severity of AD (Reitamo et al., 1998).

### KAJD Decreases Serum IgE and Inflammatory Cytokine Levels in DNCB-Treated Mice

**Figure 5:**
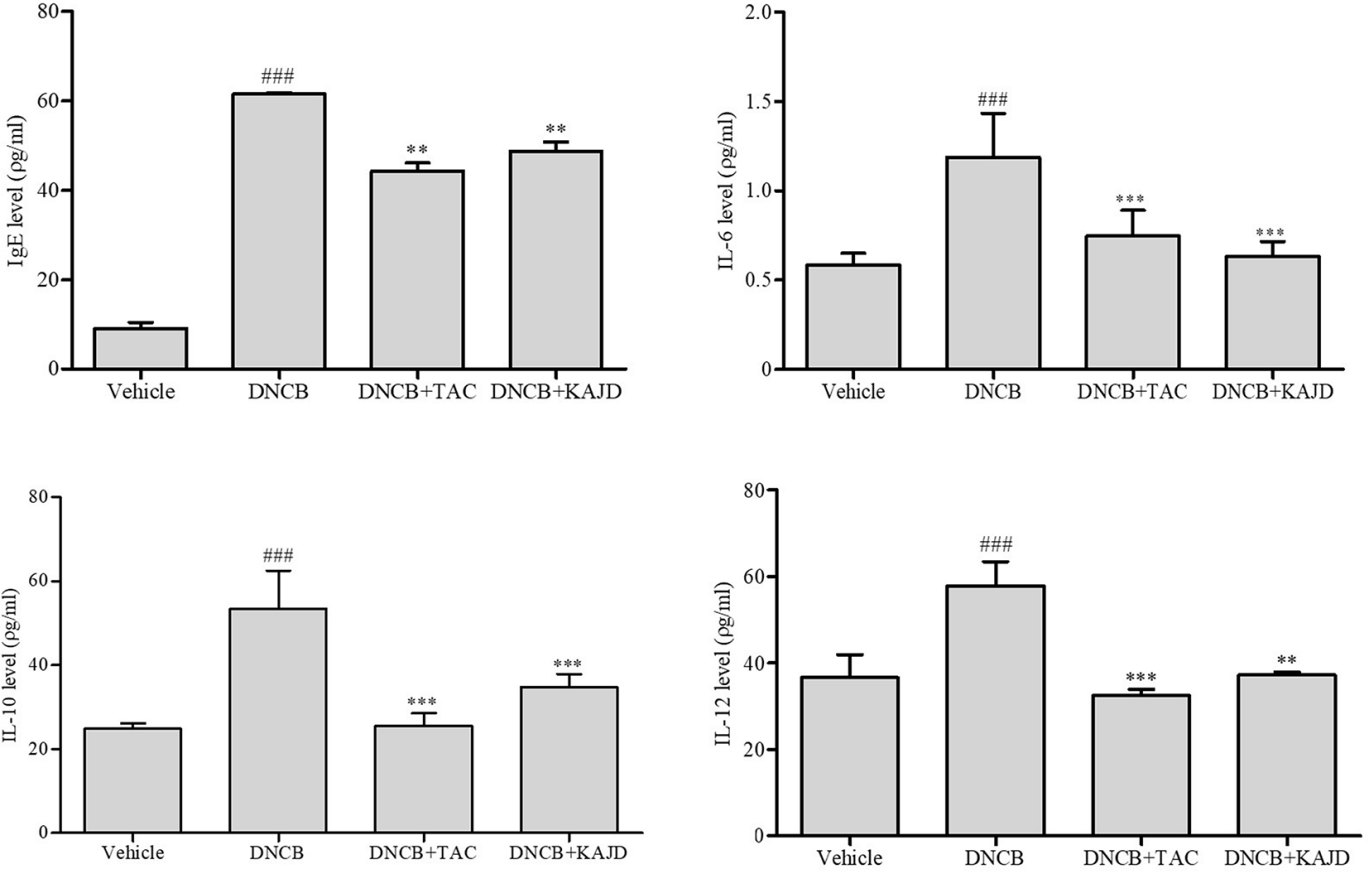
KAJD decreases the levels of serum IgE and proinflammatory cytokines. Blood samples were collected from each mouse, and the levels of serum IgE and cytokines IL-6, IL-10, and IL-12 were quantified by ELISA. ^###^ P < 0.001 compared to the vehicle group. **P < 0.01 and ***P < 0.001 compared to the DNCB-stimulated group. Vehicle, no treatment; DNCB, 2,4-dinitrochlorobenzene; TAC, tacrolimus.

To understand the mechanisms underlying the anti-inflammatory activity of KAJD, we examined its effects on the production of serum IgE and several proinflammatory cytokines in the blood of mice by ELISA, as a high serum IgE level is a major characteristic of AD. The DNCB application increased the IL-6, IL-10, and IL-12 levels, with a major increase in the serum IgE level. However, KAJD and TAC significantly suppressed the production of inflammatory cytokines and serum IgE (**Figure 5**). Note that KAJD was more effective at suppressing the DNCB-induced production of IgE and IL-6 than TAC. These results suggest that KAJD suppresses skin inflammation by inhibiting the DNCB-stimulated increase in serum IgE and proinflammatory cytokine concentrations.

### KAJD Suppresses the Agonist-Induced Production of Cytokines and Inhibits the MAPK Pathway in Several Immune Cell Types

**Figure 6:**
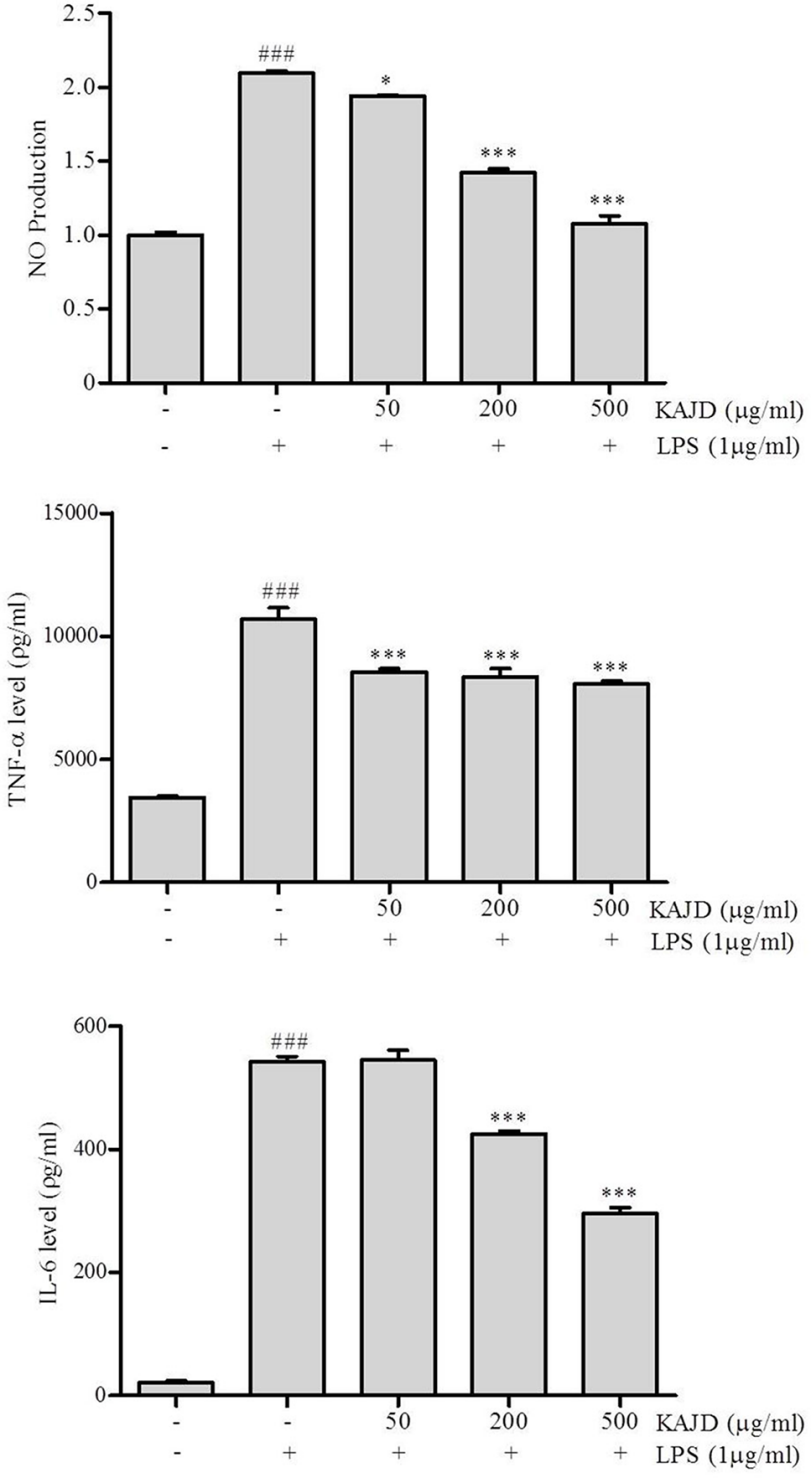
KAJD inhibits LPS-induced nitric oxide (NO) production and proinflammatory cytokine expression in RAW264.7 cells. RAW264.7 cells were stimulated with 1 μg/ml LPS for 1 h and treated with 50, 200, or 500 μg/ml KAJD for 24 h. The amount of NO in the cell culture supernatant was measured using Griess reagent. The levels of IL-6 and TNF-α in culture supernatants were measured by ELISA. Data are presented as the mean ± SEM. ^###^P < 0.001 compared to nonstimulated cells. *P < 0.05 and ***P < 0.001 compared to stimulated cells. LPS, lipopolysaccharide.

**Figure 7:**
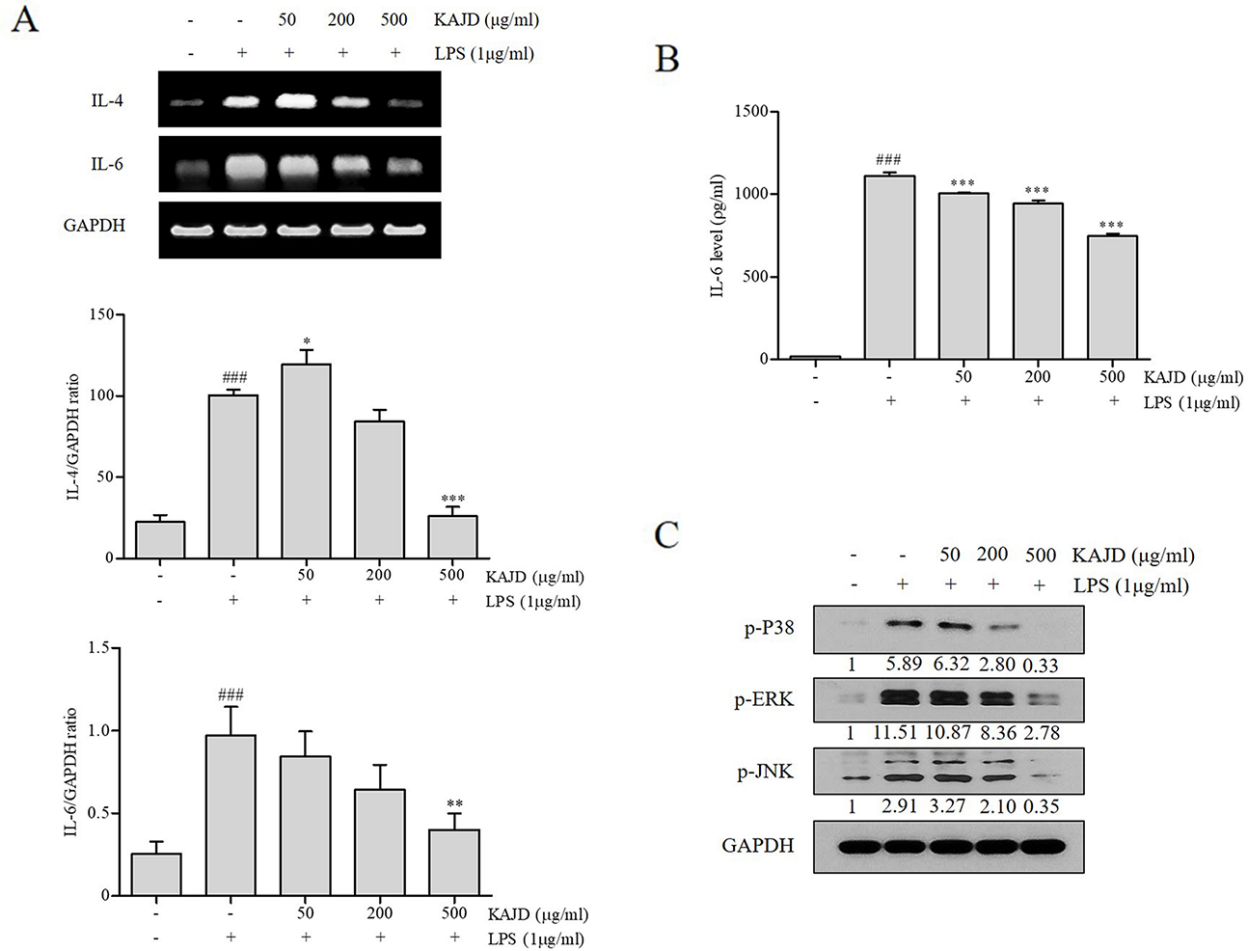
KAJD inhibits MAPK pathways and suppresses the mRNA and protein expression of proinflammatory molecules in splenocytes. **(A)** The IL-4 and IL-6 mRNA levels in splenocytes were measured by RT-PCR analysis. The bar graphs represent the quantitation of RT-PCR data. **(B)** The level of IL-6 in cell culture supernatants was measured by ELISA. Splenocytes stimulated with LPS for 1 h were treated with varying concentrations of KAJD for 24 h. **(C)** Phosphorylated ERK1/2, p38, and JNK levels in cell lysates were determined by immunoblot analysis. GAPDH was used as an internal control. Data are presented as the mean ± SEM. ^###^P < 0.001 compared to nonstimulated cells. *P < 0.05, **P < 0.01, and ***P < 0.001 compared to stimulated cells. LPS, lipopolysaccharide.

**Figure 8:**
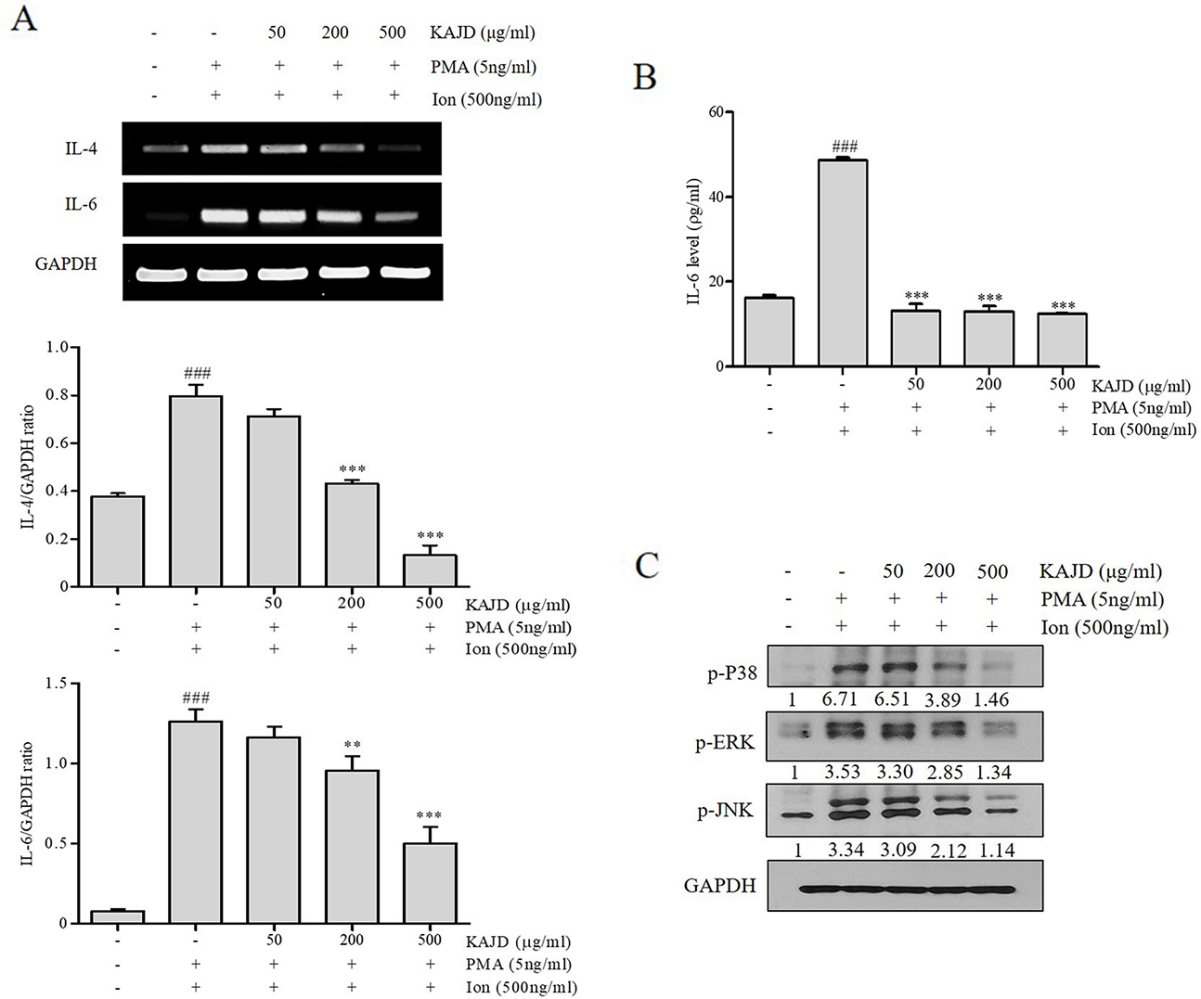
KAJD inhibits MAPK pathways and suppresses the mRNA and protein expression of proinflammatory molecules in HMC-1 cells. **(A)** The IL-4 and IL-6 mRNA levels were measured by RT-PCR in HMC-1 cells treated with the reagents described in **Figure 7** **(A)**. The bar graphs represent the quantitation of RT-PCR data. **(B)** The level of IL-6 in cell culture supernatants was measured by ELISA. HMC-1 cells were stimulated with both 5 ng/ml PMA and 500 ng/ml Ion for 1 h and treated with varying concentrations of KAJD for 24 h. **(C)** Phosphorylated ERK1/2, p38, and JNK levels in cell lysates were determined by immunoblot analysis. GAPDH was used as an internal control. Data are presented as the mean ± SEM. ^###^P < 0.001 compared to nonstimulated cells. *P < 0.05, **P < 0.01 and ***P < 0.001 compared to stimulated cells. PMA, phorbol-12-myristate-13-acetate; Ion, ionomycin.

Given the marked inhibitory effects of KAJD on cytokine production in mice, we next examined its effect on the expression of proinflammatory cytokines in RAW264.7 murine macrophage cells. LPS-mediated stimulation of RAW264.7 cells significantly promoted the expression of IL-6 and TNF-α and NO production. However, the addition of KAJD significantly reduced the expression of these cytokines and NO production in a dose-dependent manner (**Figure 6**). Moreover, we examined the role of KAJD in the suppression of cytokine expression in other immune cell types, including isolated mouse splenocytes and human mast cells (HMC-1). Similar to RAW264.7 cells, KAJD cotreatment significantly reduced agonist-stimulated IL-6 production in both cell types (**Figures 7B** and **8B**). We further tested whether KAJD regulates the expression of cytokines at the transcriptional level (**Figures 7A** and **8A**). RT-PCR analysis showed that both the IL-4 and IL-6 mRNA levels were induced by agonist treatment and decreased by KAJD cotreatment in both splenocytes and HMC-1 cells. Specifically, KAJD treatment at a concentration of 500 μg/ml reduced both IL-4 and IL-6 to the control levels. Next, we examined the viabilities of several immune cell types (RAW264.7, HMC-1, and splenocytes) following treatment with various concentrations of KAJD (**Supplementary Figures 2**–**4**). KHU-ATO-JIN-D did not induce any cytotoxicity in several immune cell types over a 24-h period. Furthermore, KAJD was shown to regulate the phosphorylation of ERK, JNK, and p38, and the inflammatory response reportedly results in the activation of MAPK pathways (Saklatvala, 2004). ERK, JNK, and p38 phosphorylation was marginally increased after treatment with 500 µg/ml KAJD (**Figures 7C** and **8C**). KHU-ATO-JIN-D regulated the phosphorylation of ERK, JNK, and p38 in a dose-dependent manner. These results suggest that KAJD suppresses proinflammatory cytokine production and inhibits MAPK pathways in several immune cell types, thereby suppressing AD-like symptoms caused by DNCB.

## Discussion

Atopic dermatitis is an inflammatory skin disease that is caused by immune dysregulation associated with various environmental and genetic factors (Schultz Larsen, 1993). The current treatments for AD include antihistamines, steroids, and immune suppressants, but side effects are very common with long-term usage of these medications (Prabhakaran et al., 1999; Simpson, 2010). The ratio and concentration of KAJD, which was used in this experiment, most suitable for cream manufacture were set after various attempts (data not shown). In this study, we showed that KAJD, herbal extracts, inhibited various AD-like skin hypersensitivity reactions, such as erythema/hemorrhage, scarring/dryness, edema, and excoriation/erosion, in BALB/c mice exposed to DNCB. Treatment with KAJD suppressed not only the infiltration of inflammatory immune cells into AD skin lesions but also the levels of several proinflammatory cytokines in the blood. Therefore, our results suggest that KAJD can be an effective treatment option for AD. Moreover, KAJD shows no toxicity and effectively attenuates AD symptoms; thus, developing this extract as a treatment for AD is important. It should be noted that KAJD performs better than TAC.

Since many reports have demonstrated that Th2-type immune responses play significant roles in the pathogenesis of AD; Th2 cytokines are considered to be attractive therapeutic targets for AD treatment. In AD skin lesions, Th2 lymphocytes produce high levels of IL-4, IL-5, IL-6, IL-10, and IL-13, which induce IgE production by B cells. IgE, in turn, binds to the high-affinity IgE-Fc receptor type I (FcϵRI) on the surface of mast cells, leading to the release of various types of inflammatory mediators, such as histamine, chemokines, and cytokines. Eosinophil recruitment to inflamed skin *via* Th2-type cytokines is another important characteristic of AD. Our findings showed that KAJD reduced the serum levels of IgE and Th2 cytokines, such as IL-6 and IL-10, and the infiltration of mast cells and leukocytes, including eosinophils and monocytes, into AD skin lesions.

In addition to Th2 cytokines, proinflammatory cytokines such as TNF-α are also known to play important roles in AD pathogenesis by disrupting skin barrier function. A recent study showed that TNF-α alone or in combination with Th2 cytokines changes the skin barrier lipid composition and epidermal morphogenesis, leading to skin barrier perturbation (Danso et al., 2014). The disrupted barrier in AD skin becomes vulnerable to the penetration of allergens through the skin. In response to these stimuli, keratinocytes are able to secrete proinflammatory mediators, which increase the recruitment and activation of T cells and dendritic cells (DCs) (Pastore et al., 2006). Moreover, keratinocytes in the disrupted skin produce thymic stromal lymphopoietin (TSLP), an epithelial cell-derived cytokine that strongly activates DCs, which prime naïve CD4+ T cells to differentiate into Th2 cells (Liu, 2009). Therefore, in AD, the skin forms a positive feedback loop between cytokines and barrier-disrupted skin with highly elevated levels of proinflammatory and Th2 cytokines. In this study, we showed that KAJD treatment suppressed both the infiltration of T cells and the expression of the TNF-α and Th2 cytokines, indicating that KAJD might inhibit some point in this positive feedback loop in AD skin.

Macrophages are also involved in skin inflammation in AD. Although macrophage activation is beneficial for eliminating inflammatory stimuli during acute inflammatory responses, persistent macrophage activation results in the development of chronic inflammatory skin diseases, including psoriasis and AD (Valledor et al., 2010). Activated macrophages produce proinflammatory cytokines, including IL-1, IL-6, IL-12, and TNF-α, and high levels of inducible nitric oxide synthase (iNOS), thus contributing to the pathogenesis of AD (Kasraie and Werfel, 2013). Similarly, Th2 cells release various cytokines, such as IL-4, IL-6, and IL-10, to induce B cells to produce IgE, which, in turn, activates mast cells for histamine release (Hammad and Lambrecht, 2008; Holgate and Polosa, 2008). Moreover, the spleen, the largest lymphatic organ in the body, acts as storage for inflammatory monocytes and differentiates into macrophages to participate in both anti-inflammatory and anti-inflammatory reactions (Auffray et al., 2007; Hiroyoshi et al., 2012; Robbins et al., 2012; Wystrychowski et al., 2014). Notably, topical application of KAJD on BALB/c mice suppressed the levels of IL-12, which were increased by exposure to DNCB (**Figure 5**). Moreover, ELISA analysis of murine macrophage RAW 264.7 cells revealed that KAJD inhibited the LPS-induced production of IL-6, TNF-α, and NO. The MAPK pathway is essential for the pathogenesis of the inflammatory response (Wu, 2004). As mentioned in previous studies, MAPK inhibition attenuates severe inflammatory responses in the dermis (Ipaktchi et al., 2006). In our attempt to investigate the molecular mechanisms by which KAJD inhibits the production of Th2 cytokines, we found that KAJD dramatically downregulates IL-4 and IL-6 mRNA expressions and inhibits the MAPK (p38, ERK, and JNK) pathways in immune cells, such as murine splenocytes and human mast cells stimulated with LPS, indicating that KAJD alleviates several AD symptoms by controlling the transcriptional expression of Th2 cytokines. However, further studies are needed to identify signaling pathways that govern the KAJD-mediated transcriptional control of these Th2 cytokines.

Notably, KAJD was more effective at alleviating several AD symptoms, including skin thickness and infiltration of mast cells and CD4+ T cells, than TAC, which was used as a control in this study. In addition, KAJD did not show cell toxicity in multiple cells at the concentration used in our experiments.

Taken together, these results demonstrate that KAJD is very effective for the treatment of AD by suppressing the recruitment of numerous inflammatory cells, the production of proinflammatory cytokines, and the MAPK pathway. Further studies are warranted to investigate the clinical usefulness of KAJD.

## Data Availability

The raw data supporting the conclusions of this manuscript will be made available by the authors, without undue reservation, to any qualified researcher.

## Ethics Statement

All the experiments were performed in accordance with the protocols approved by the Institutional Animal Care and Use Committees of Kyung Hee University (Approval Number KHUASP (SE)-14-014). The authors highly acknowledged the Department of Preventive Medicine for providing lab facilicities.

## Author Contributions

SH carried out the experiment and drafted the manuscript. SH, JK and HK revised the research and manuscript and assisted in the research work. SH, TK, HS and YS guided the research and revised and submitted the manuscript. S-GK supervised the research. All the authors read and approved the final manuscript.

## Funding

This research was supported by a grant from the Korean Medicine R&D Project of the Ministry of Health and Welfare (HI12C1889 and HI13C0530, HI11C2110). The funding sponsors had no role in the study design, performance, data collection and analysis, decision to publish, or preparation/writing of the manuscript.

## Conflict of Interest Statement

The authors declare that the research was conducted in the absence of any commercial or financial relationships that could be construed as a potential conflict of interest.

## Abbreviations

PUL, Pyrus ussuriensis Maxim. Leaves; AD, Atopic dermatitis; NO, Nitric oxide; IL-6, Interleukin 6; IL-1β, Interleukin 1β; TNF-α, tumor necrosis factor; DNCB, 2, 4-dinitrochlorobenzene; IgE, Immunoglobulin E; IL-4, Interleukin 4; IL-13, Interleukin 13; HPLC, High performance liquid chromatography; TEWL, Transepidermal water loss; DMEM Dulbecco’s modiﬁed Eagle’s medium; FBS, Fetal bovine bovine bovine serum; LPS, Lipopolysaccharide; DMSO, Dimethyl sulfoxide; PBS, phosphate-buﬀered saline; H&E, Hematoxylin and eosin; SEM, Standard error error of the mean; ANOVA, Analysis of variance.

## Supplementary Material

Figure S1 Changes in **(A)** mouse body weight and **(B)** food intake during treatment with KAJD. Values are expressed as the mean ± SEM (n=8). Vehicle, no treatment; DNCB, 2,4-dinitrochlorobenzene; TAC, tacrolimus.

Figure S2 Effect of KAJD on LPS-stimulated RAW264.7 cell viability. RAW264.7 cells were treated with various concentrations of KAJD for 24 h after vehicle (upper panel) or LPS (lower panel) pretreatment for 1 h (upper panel). After treatment, cell viability was measured using a WST assay. Data are presented as the mean ± SEM. ^###^P< 0.001 compared to nonstimulated cells. ***P < 0.001 compared to stimulated cells. LPS, lipopolysaccharide.

Figure S3 Effect of KAJD on LPS-stimulated splenocyte cell viability. Splenocytes were treated with various concentrations of KAJD for 24 h after vehicle (upper panel) or LPS (lower panel) pretreatment for 1 h (upper panel). After treatment, cell viability was measured using a WST assay. Data are presented as the mean ± SEM. ^##^P < 0.01 and ^###^P < 0.001 compared to nonstimulated cells. **P < 0.01 compared to stimulated cells. LPS, lipopolysaccharide.

Figure S4 Effect of KAJD on PMA and ionomycin-stimulated HMC-1 cell viability. HMC-1 cells were treated with various concentrations of KAJD for 24 h after the pretreatment of vehicle (upper panel) or PMA plus Ion (lower panel) for 1 h (upper panel). After treatment, cell viability was measured using a WST assay. Data are presented as the mean ± SEM. ^###^P < 0.001 compared to nonstimulated cells. *P < 0.05 and ***P < 0.001 compared to stimulated cells. PMA, phorbol-12-myristate-13-acetate; Ion, ionomycin.
