# Supplementary figures and images for: Topical Application of KAJD Attenuates 2,4-Dinitrochlorobenzene-Induced Atopic Dermatitis Symptoms Through Regulation of IgE and MAPK Pathways in BALB/C Mice and Several Immune Cell Types
Source: Front Pharmacol. 2019 Sep 19;10:1097. doi: 10.3389/fphar.2019.01097 (PMC6761305; doi:10.3389/fphar.2019.01097)

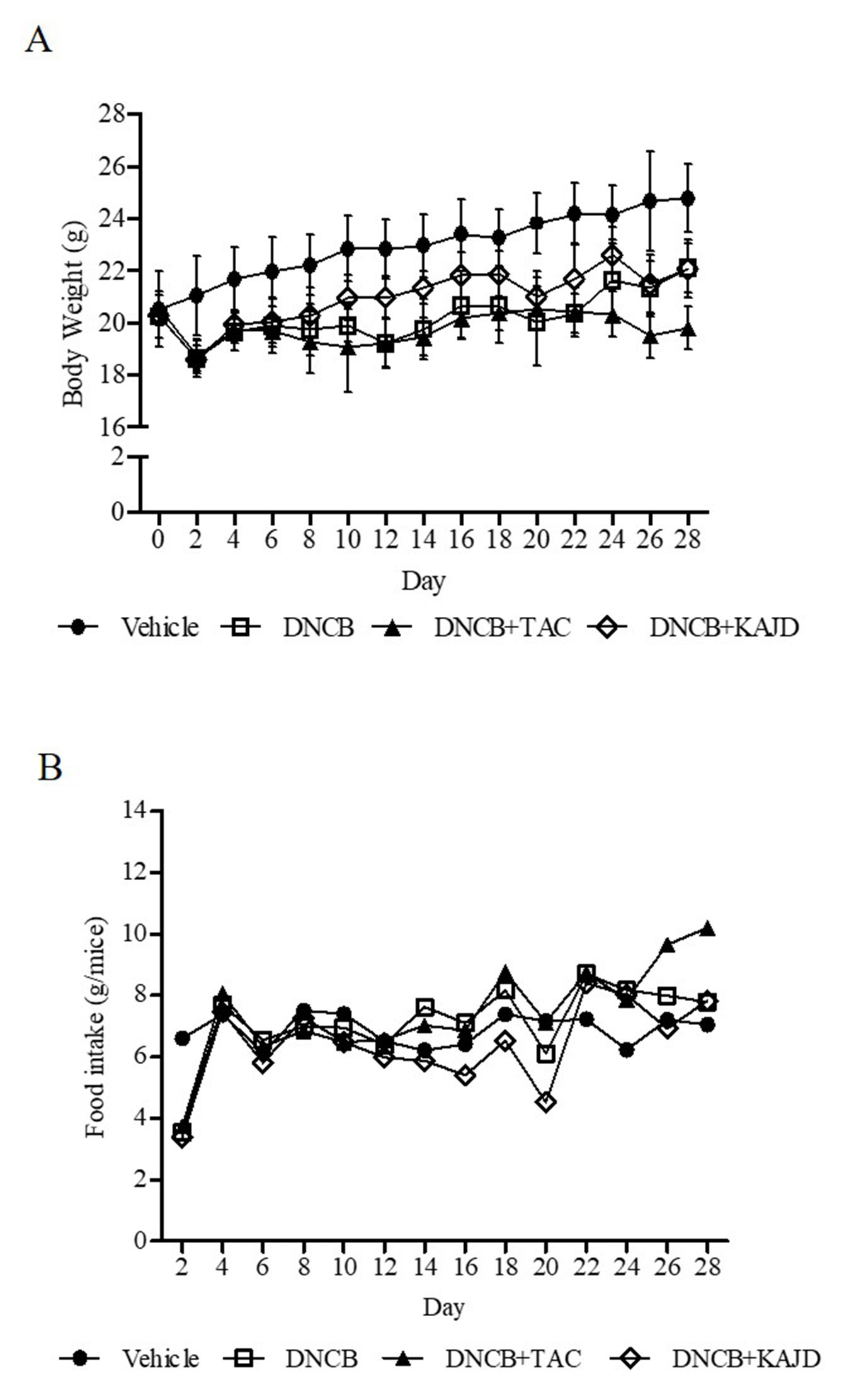

Supplement: Figure S1 — Changes in (A) mouse body weight and (B) food intake during treatment with KAJD. Values are expressed as the mean ± SEM (n=8). Vehicle, no treatment; DNCB, 2,4-dinitrochlorobenzene; TAC, tacrolimus. [file Image_1.jpeg]

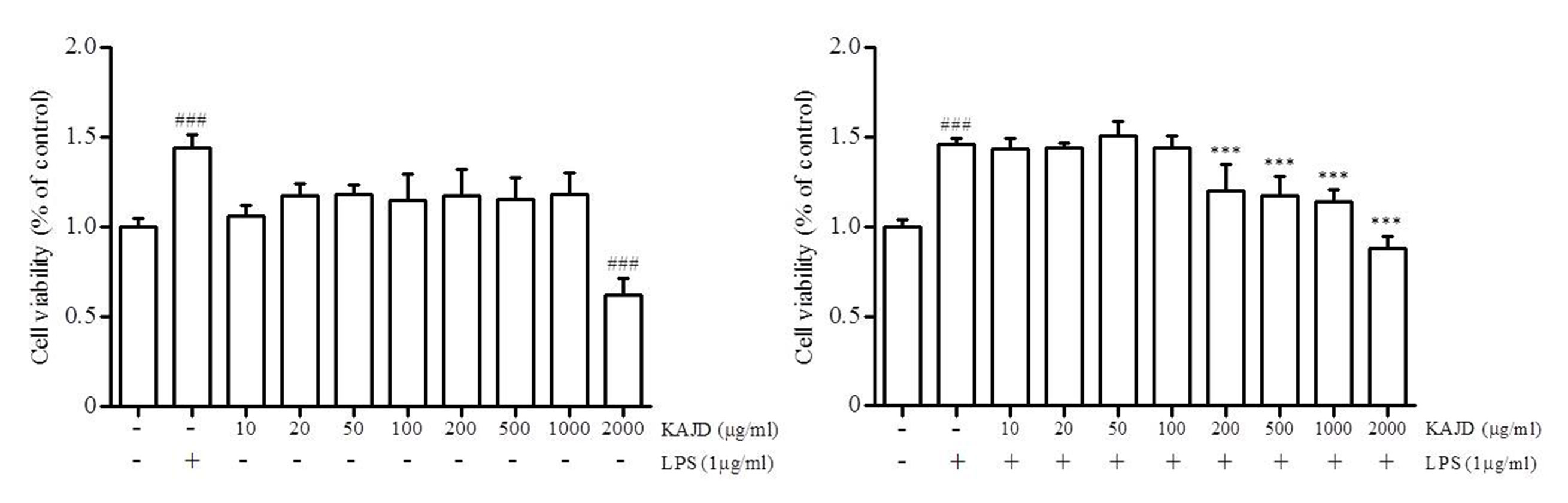

Supplement: Figure S2 — Effect of KAJD on LPS-stimulated RAW264.7 cell viability. RAW264.7 cells were treated with various concentrations of KAJD for 24 h after vehicle (upper panel) or LPS (lower panel) pretreatment for 1 h (upper panel). After treatment, cell viability was measured using a WST assay. Data are presented as the mean ± SEM. ###P< 0.001 compared to nonstimulated cells. ***P < 0.001 compared to stimulated cells. LPS, lipopolysaccharide. [file Image_2.jpeg]

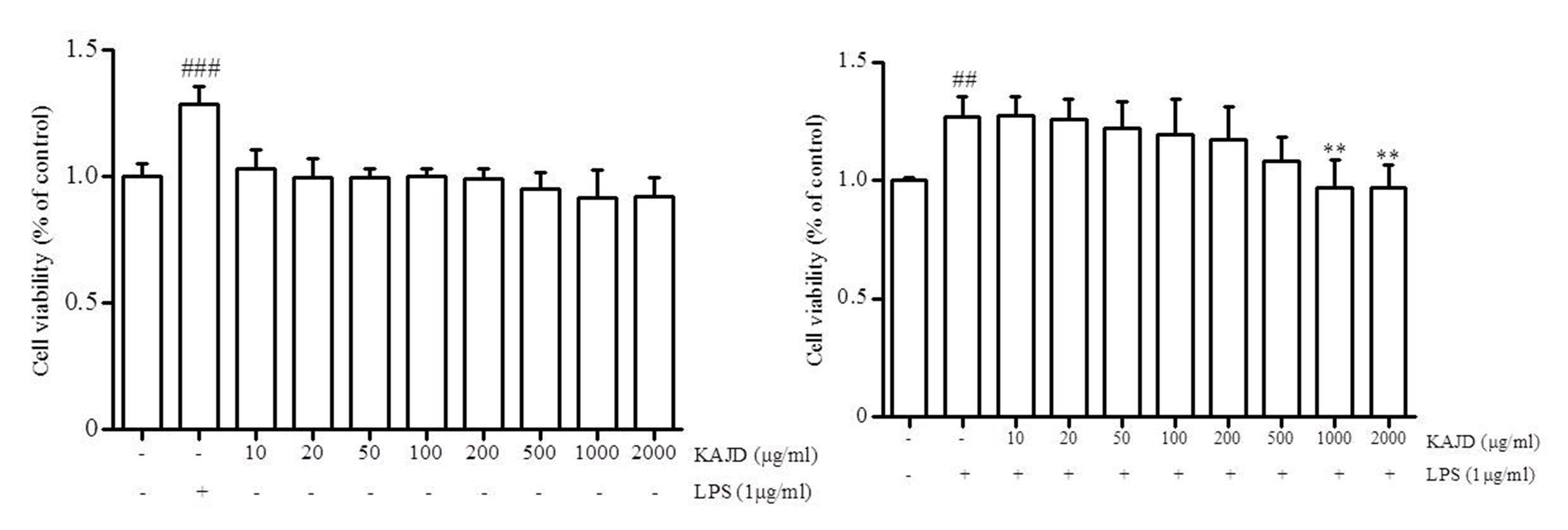

Supplement: Figure S3 — Effect of KAJD on LPS-stimulated splenocyte cell viability. Splenocytes were treated with various concentrations of KAJD for 24 h after vehicle (upper panel) or LPS (lower panel) pretreatment for 1 h (upper panel). After treatment, cell viability was measured using a WST assay. Data are presented as the mean ± SEM. ##P < 0.01 and ###P < 0.001 compared to nonstimulated cells. **P < 0.01 compared to stimulated cells. LPS, lipopolysaccharide. [file Image_3.jpeg]

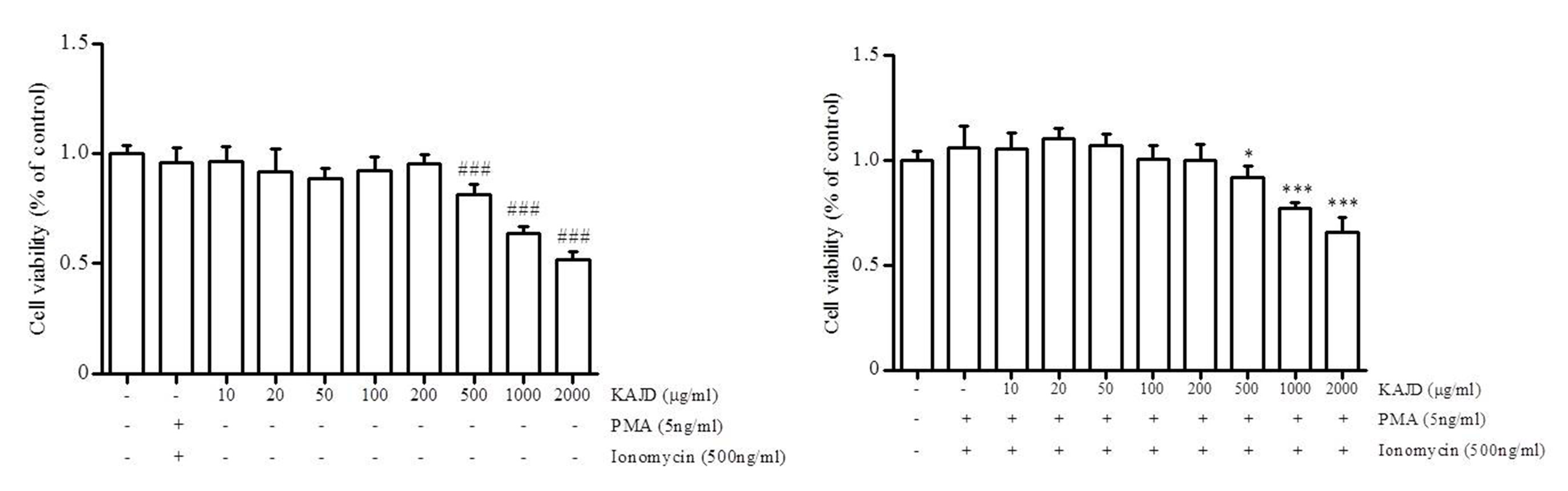

Supplement: Figure S4 — Effect of KAJD on PMA and ionomycin-stimulated HMC-1 cell viability. HMC-1 cells were treated with various concentrations of KAJD for 24 h after the pretreatment of vehicle (upper panel) or PMA plus Ion (lower panel) for 1 h (upper panel). After treatment, cell viability was measured using a WST assay. Data are presented as the mean ± SEM. ###P < 0.001 compared to nonstimulated cells. *P < 0.05 and ***P < 0.001 compared to stimulated cells. PMA, phorbol-12-myristate-13-acetate; Ion, ionomycin. [file Image_4.jpeg]
